# Comparison of the precision of smooth pursuit in humans and head unrestrained monkeys

**DOI:** 10.16910/jemr.11.4.6

**Published:** 2018-11-09

**Authors:** Jan Churan, Doris I. Braun, Karl R. Gegenfurtner, Frank Bremmer

**Affiliations:** University of Marburg & CMBB, Marburg, Germany; Giessen University & CMBB, Giessen, Germany

**Keywords:** Eye movement, eye tracking, saccades, smooth pursuit, non-human primates, head unrestrained

## Abstract

Direct comparison of results of humans and monkeys is often complicated by differences in experimental conditions. We replicated in head unrestrained macaques experiments of a recent study comparing human directional precision during smooth pursuit eye movements (SPEM) and saccades to moving targets (Braun & Gegenfurtner, 2016). Directional precision of human SPEM follows an exponential decay function reaching optimal values of 1.5°-3° within 300 ms after target motion onset, whereas precision of initial saccades to moving targets is slightly better. As in humans, we found general agreement in the devel-opment of directional precision of SPEM over time and in the differences between direc-tional precision of initial saccades and SPEM initiation. However, monkeys showed over-all lower precision in SPEM compared to humans. This was most likely due to differences in experimental conditions, such as in the stabilization of the head, which was by a chin and a head rest in human subjects and unrestrained in monkeys.

## Introduction

Non-human primates (NHPs) serve as animal model for the investigation of the neural basis of eye movements (1, 2, 3, 4, 5, 6, 7). Humans and NHPs share many properties of their visual and oculomotor systems (8, 9, 10, 11, 12, 13, 14). Also, psychophysical studies showed that many aspects of visual perception are remarkably similar in both species (15 16 17). Due to the retinal architecture with high resolution processing only in the fovea, primates move their eyes typically 2-3 times per second. Voluntary eye movements, saccades and smooth pursuit eye movements (SPEM), are the means by which humans and NHPs bring the projection of potentially interesting objects onto the fovea and keep them in place despite relative motion between the observer and the target object. Traditionally it was assumed that saccades and smooth pursuit were generated by distinct cortical and subcortical networks and specific paradigms were used to study them in isolation. More recently, however, many saccade-pursuit interactions have been found at the neuronal and behavioral level (see 18, 19) and several studies showed how and when saccades and SPEMs interact (e.g. 20, 21, 22, 23, 24, 25, 26). The comparison of results gained in humans and monkeys, however, typically becomes complicated by different experimental approaches. In human oculomotor studies, experimental setups often employ a chin- and/or headrest or a bite bar in order to stabilize the subjects’ head and allow for stable eye movement recordings. NHPs, however, are studied typically in a head-fixed preparation to allow for concurrent neurophysiological recordings. The question remains if and how such head-immobilization influences oculomotor behavior. It is rare that exactly the same paradigm is used for human and monkey observers. Therefore, we investigate here the influence on the relationship of the directional precision of pursuit and initial saccades.

In everyday life, saccades and SPEM are often combined, for example when we try to follow a moving ball during a tennis or soccer match. In this situation, we first initiate a saccade to foveate rapidly the ball and then try to follow its movements by SPEM. Previously, the time course of pursuit precision has been investigated mainly in a so called step-ramp paradigm that avoids the initial saccadic eye movements. In humans, directional precision of SPEM in the step-ramp paradigm follows an exponential decay function that reaches optimal values between 1.5°-3° within 300 ms after target motion onset (27, 28, 29, 30). In head-restrained monkeys, pursuit thresholds for direction reach values < 2-3° quite similar to perceptual threshold of direction discrimination during fixation (7). Directional precision in a paradigm with initial saccades to moving targets, however, was so far only tested in Braun & Gegenfurtner (30).

When we scan our surrounds, we coordinate eye and head movements and generate gaze saccades to displace rapidly our visual axis in space. Gaze saccades require the coordination of both mobile segments, i.e. head and eyes. However, the precision and accuracy of gaze saccades are comparable to that of eye saccades made with the head fixed (31, 32, 33, 34, 35). For SPEM in combined eye-head movements one may expect changes in the precision of gaze since here the pursuit mechanism must be combined with head-movement commands and vestibular signals (36, 37) both of which may contribute to the variability of SPEM (28). The central goal of our study was to measure the directional precision of saccades and SPEMs in the head unrestrained macaque and to provide further evidence for the validity of the macaque as animal model for human visuo-motor processing. To this end, we replicated the experiments recently conducted in humans (30) and determined in head-unrestrained monkeys the dynamics of directional precision of SPEM and initial saccades to moving targets. Our main questions were (1) whether the overall structure of the increase in SPEM precision over time is similar in humans and in head unrestrained NHPs, (2) whether there are similar differences between the precision during SPEM and the precision of initial saccades in the two species and (3) whether there are major general differences in the performance that might be attributed to the head-unrestrained setting in which the NHPs were tested.

## Methods

### Subjects

Two male adult macaque monkeys, monkey ME and monkey MB participated in the experiment. Both animals were well trained by other oculomotor experiments and used to the test conditions. All procedures had been approved by the regional ethics committee and were in accordance with the published guidelines on the use of animals in research (European Communities Council Directive 2010/63/EU).

### Apparatus and test conditions

**Figure 1:**
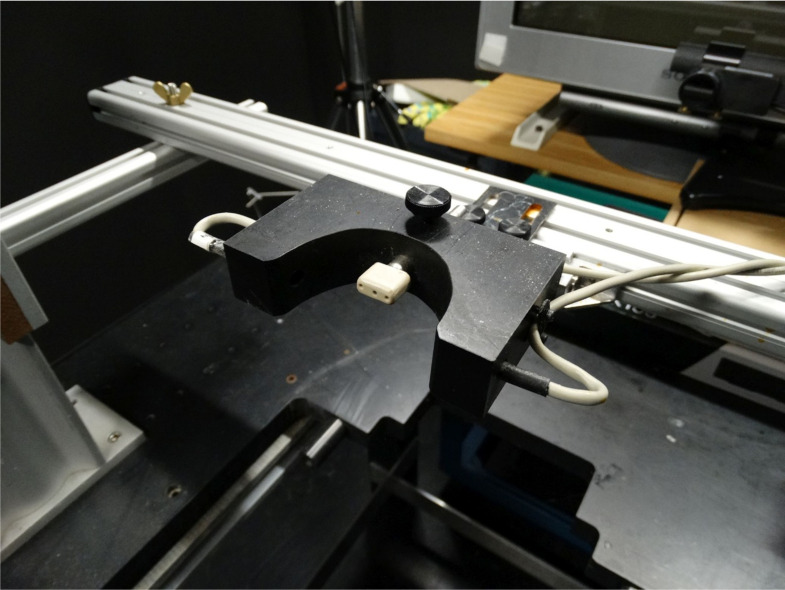
Experimental set-up. The mouthpiece (white) of the reward system and the photoelectric barrier (within the black block with the half-circular opening) to detect whether the monkey’s head was in a position suitable for the measurement of eye movements are in front, the monitor and the camera for eye movement measurements are in the back.

Directional precision of saccades and SPEM was measured with their heads freely moveable and unrestricted (Figure 1). The monkey was seated in a conventional primate chair in front of a monitor. Eye movements of the right eye were recorded with an infrared video-oculographic system (EyeLink 1000, SR Research Ltd., Osgoode, Canada) running at 1000 Hz. Testing only could be performed when the monkey’s head was in the appropriate position for the video camera system to detect the monkey’s right eye. To encourage the monkey to place its head in a suitable position for eye movement measurements and execution of the oculomotor task, we combined our reward system with a custom made mouth piece as shown in Figure 1. The mouth piece contained a photoelectric barrier and only when the monkey`s mouth interrupted the light beam, an experimental trial was started. This mouthpiece allowed for some variability of head orientations that could move by about +/-15° around the yaw axis and about +/- 10° around the pitch axis without interrupting the trial (a drawing of the mouthpiece can be obtained from the corresponding author on reasonable request).

Stimuli were presented on a color monitor (Sony Trinitron GDM F520, resolution 1280 x 1024 pixels) placed 60 cm in front of the monkey. The display subtended 37 x 28 degrees of the central visual field. All stimuli were generated using the Psychophysics Toolbox (38, 39, 40).

### Ramp and Step-Ramp paradigms

**Figure 2:**
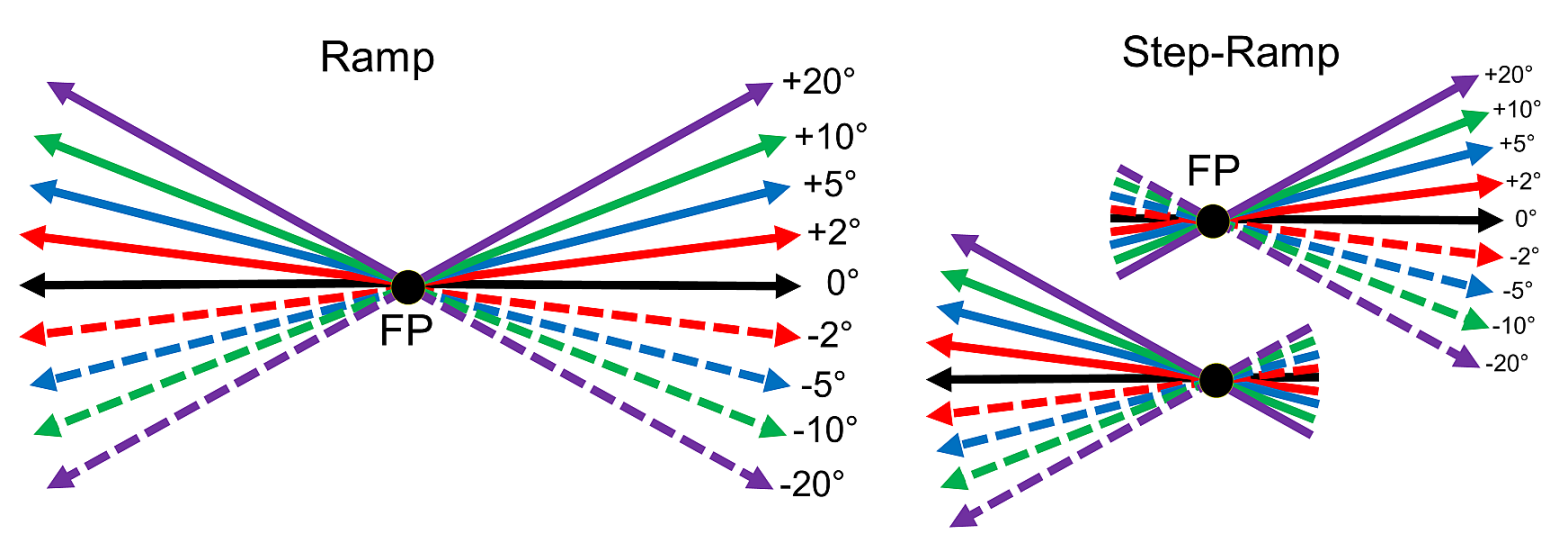
Diagram of the stimuli used to measure the directional precision of initial saccades and smooth pursuit (after Braun & Gegenfurtner, 2016). Left: In the Ramp paradigm, the eye movement target moves randomly after an initial fixation period left- or rightward at 10°/s. No or one out of eight different vertical components of +/- 20°, 10°, 5°, or 2° was added unpredictably to the horizontal ramp direction. Right: In the Step-Ramp paradigm after initial fixation the target first makes a step contraversive to the direction of the upcoming ramp motion in one of the indicated directions. Different colors represent different vertical components, solid lines represent upward- and dashed lines downward vertical components.

We tested the directional precision of initial saccades and SPEM to ramp target movements very similar to two paradigms recently tested in human subjects (30). In the ramp paradigm first a small red fixation spot was presented in the center of a uniform gray screen (38 cd/m^2^) for a randomized duration between 500-1000 ms (see Ramp paradigm, Figure 2). When the monkey kept its head in the appropriate position, i.e. interrupting the beam of the light barrier with its mouth, and fixated the central spot for 500-800 ms, the fixation spot was replaced by a pursuit target that moved immediately at a constant speed of 10°/s randomly either leftward or rightward across the screen for 1 s. One out of nine different vertical components of 0°, ±2°, ±5°, ±10° and ±20° was added unpredictably to the horizontal direction. In this paradigm, the monkey made first a target directed initial saccade and followed then the moving target with SPEM.

The second paradigm was the classical step-ramp paradigm developed by Rashbass (41) to elicit pure SPEMs without initial saccades. Here, the only difference compared to the ramp condition was, that the pursuit target was displaced by a small step in the direction contraversive to the direction of the upcoming target motion (Step-Ramp paradigm, Figure 2). In this paradigm, the contraversive step eliminates the necessity for an initial saccade. The step size of the pursuit target was adjusted for each monkey to minimize the occurrence of saccades during the initiation phase of pursuit. For both monkeys the best step size was 1.5 deg. After each trial, the monkey was rewarded for keeping the eye position within a 7° window around the fixation and the pursuit target by a drop of water. The ramp and step-ramp paradigms were presented in separate blocks. A single block lasted for approximately 1 hr and the monkey usually achieved 400-700 trials in each block. The order of the blocks was pseudo-random.

### Data processing and eye movement analysis

Typically, several hundred trials were collected for each paradigm (ramp and step-ramp) and each vertical ramp component. All data processing was done using the MATLAB (MathWorks, Natick, USA) programming package. Trials were excluded if any saccade was detected in a time-window from 200 ms before to 500 ms after stimulus motion onset during step-ramp trials. In ramp trials only one saccade was permitted, the latency of which had to be 100 ms to 300 ms from the onset of the stimulus motion. Due to these strict exclusion criteria, ~50% of the trials were rejected from further analysis in each of the conditions. On average, 657 successful trials remained in the Ramp paradigm for each vertical component in monkey MB (altogether 5912 trials) and 224 trials for each vertical component for monkey ME (altogether 2015 trials). In the Step-Ramp paradigm on average 426 trials were successful in each condition for monkey MB (3831 trials) and 311 trials for monkey ME (2796 trials).

To quantify directional precision during SPEM as well as during initial saccades, we constructed oculometric functions for each point in time (42, 43) using similar methods as described in more detail in Braun & Gegenfurtner (30). This method allowed us to compute the temporal profile of the directional precision of pursuit to step-ramp targets and of initial saccades to ramp targets.

In short, for pure SPEM to step-ramp stimuli, we first calculated the vertical and horizontal eye velocities in 1 ms time bins and smoothed the resulting speed profiles using a running average with a window size of 40 ms. We aligned the velocity traces for the step-ramp trials to the SPEM onset. SPEM onsets were calculated in each trial using the velocity profile in a time window of 10 ms that was centered on the point in time when the eye speed first exceeded 5°/s. A regression line was fitted to the velocity trace in that time window and the intersection of the regression line with x-axis was used to determine the SPEM onset (44, 45). The algorithm failed in ~9% of the cases in each monkey and those trials were excluded from further analysis.

**Figure 3:**
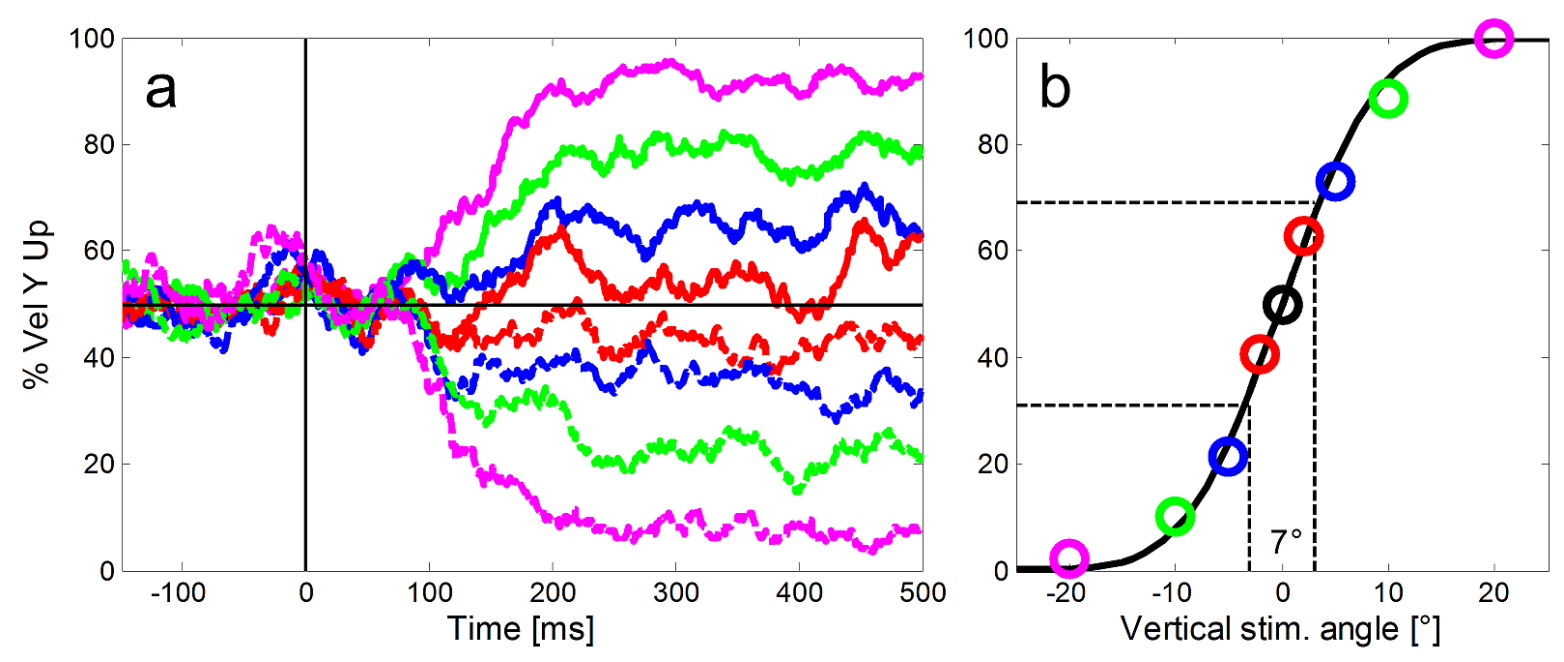
Illustration of our method to calculate the directional precision of SPEM in time bins of 1 ms. a: For the calculation of the directional precision we used all step-ramp trials collected from each monkey (here example data from monkey ME). For each ramp direction (shown here in different colors and line types as introduced in Figure 2), we determined the proportions of trials with an upward eye velocity (y-axis) relative to the horizontal baseline (black horizontal line). Shortly after 100 ms SPEM started to deviate according to the ramp direction of the target. b: In a second step a cumulative Gaussian was fitted to the proportions of upward eye movements to estimate the directional precision at each point in time (color codes of the single markers represent different vertical components of the stimulus as introduced in Figure 2). The slope of the function - quantified as the difference of vertical stimulus angles that was required to reach 31% and 69% upward responses (indicated by the dashed black lines) was used to measure the precision of the eye movements. Here the directional threshold was 7°.

To remove any directional bias, we used the median vertical eye velocity in response to purely horizontal ramp movements (black line in Figure 3a) as baseline for data from each monkey. For the eight step-ramp directions that had a non-zero vertical component we subtracted the vertical eye speed component from this baseline. In a next step, we calculated for each of the eight step-ramp directions the proportion of trials with an upward vertical eye direction for each 1 ms time bin (Figure 3a). Then we fitted a cumulative Gaussian to the proportions of upward trials (Figure 3b) to estimate the directional precision of the eye responses at the selected point in time. We chose the difference between the 69% and the 31% points of the Gaussian, irrespective of lapse and guess rates, as our estimate of the directional oculometric thresholds. This procedure led to more robust threshold estimates than simply taking the standard deviation of the Gaussian. Note that due to this calculation, low numeric values of the thresholds indicate high directional precision, i.e. better performance. To estimate the time course of the increase of directional precision for pure SPEM, we used the least squares method to fit an exponential decay function to the time course of threshold data (function ‘nlinfit’ in MATLAB):

**(1):** P(t)=x_1_+x_2_*exp(-x_3_/t)

Where x_1_ is the asymptote of the function, x_2_ is the scaling factor and x_3_ represents the time constant of the decay function.

### Directional precision of initial saccades to moving targets

We calculated the directional precision for the whole time course of SPEM in the step-ramp paradigm. For data from the ramp paradigm we mainly focused on the directional precision during the initial saccade since our aim was to compare the directional precision of pure SPEM responses to the precision of the first initial saccade during the same time interval. To measure the directional precision of initial saccades to ramp targets we aligned the eye traces of saccades to their onsets and constructed oculometric functions based on the average vertical component added to the median direction of the eye in response to purely horizontal ramps. Peri-saccadic direction thresholds were averaged in a 30 ms time window beginning from the saccade onset. A time window of 30 ms was chosen because it was the average duration of the initial saccades.

## Results

By measuring the oculometric directional thresholds for each point in time we could study the development and dynamics of the directional precision of the pursuit system.

**Figure 4:**
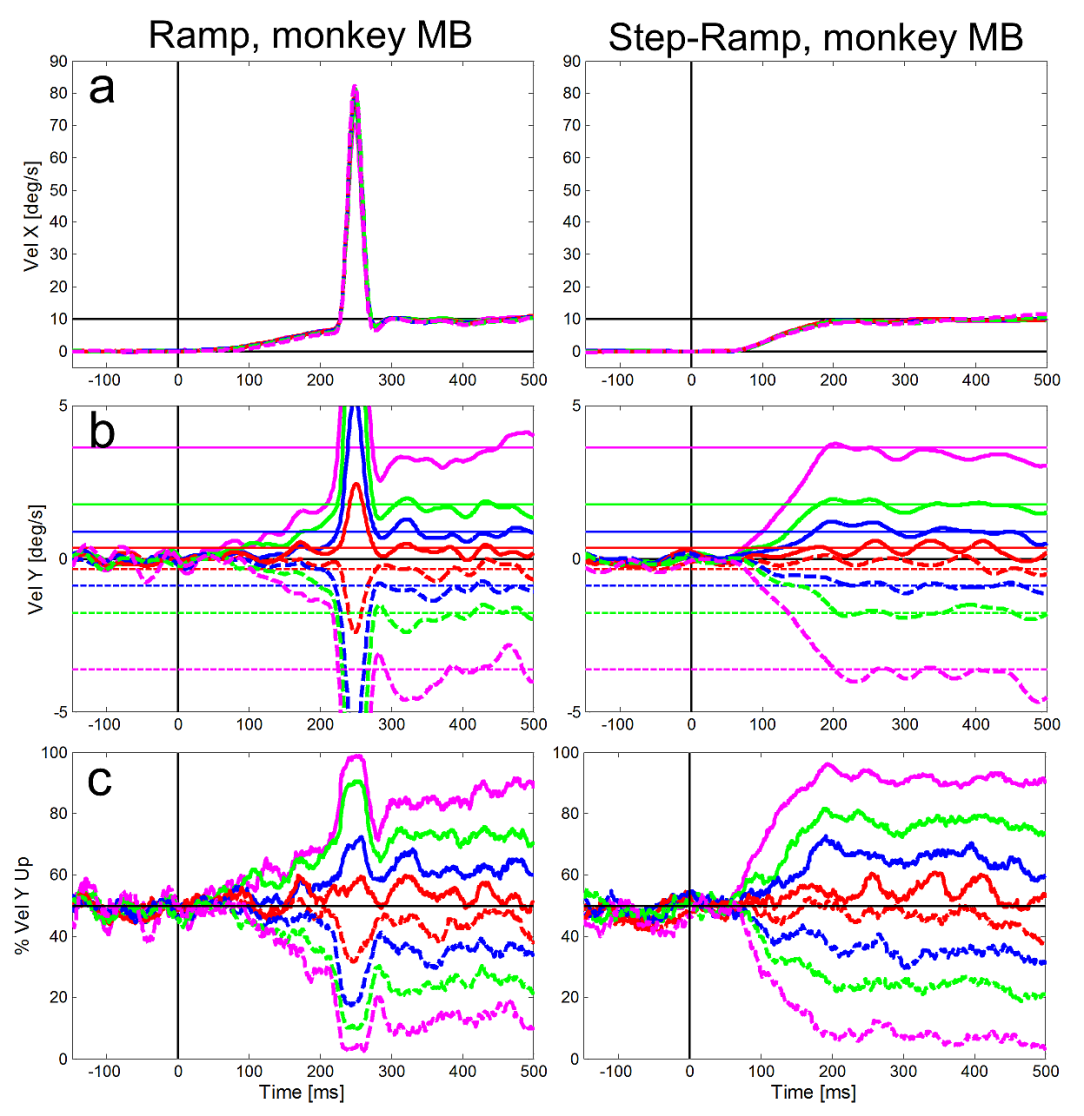
Averaged eye velocities for the ramp (left column) and step-ramp (right column) paradigms for monkey MB. The different vertical stimulus components are coded by different line colors as shown in in Figure 2. Like in Figure 2, solid lines represent upward stimulus movements, while dashed lines represent downward stimulus movements the vertical components are 2° (red), 5° (blue), 10° (green) and 20° (magenta). a: Horizontal eye velocities, b: Vertical eye velocities, the vertical target velocities are indicated by thin horizontal lines in the respective color. c: Percentages of trials (y-axis) in which the vertical eye velocity was ‘upward’. All eye velocity traces are baseline corrected for the eye movement to pure horizontal target motion.

**Figure 5:**
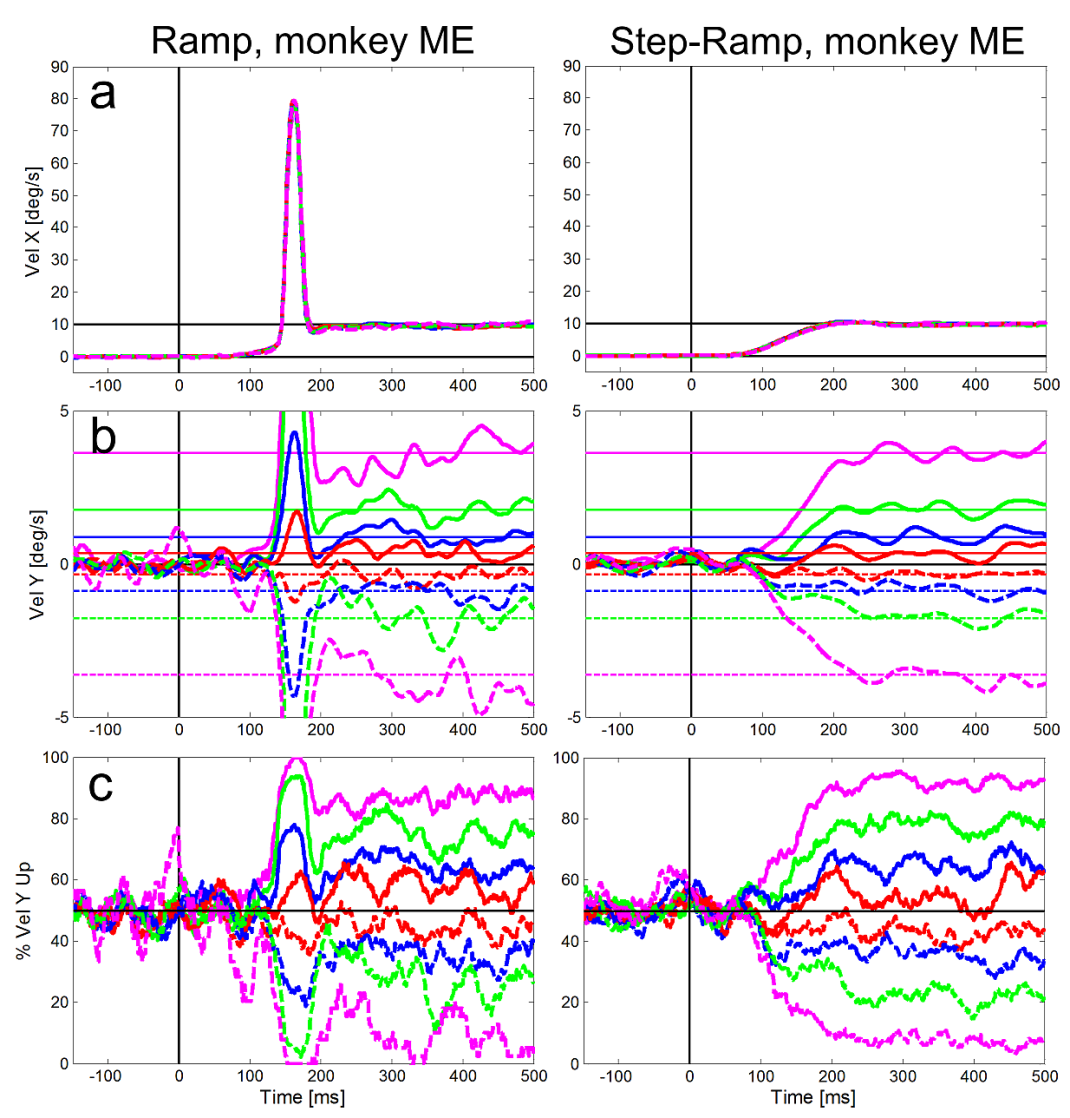
Averaged eye velocities for the ramp (left column) and step-ramp (right column) paradigms for monkey ME. All conventions like in Figure 4.

Figures 4 and 5 show for our two monkeys separately the averaged eye velocities of initial saccades and pure SPEM measured with the ramp-paradigm (left column) and the step-ramp paradigm (right column). A similar plot for the step-ramp paradigm with human subjects can be found in Braun & Gegenfurtner (30) in their figure 2B. Both, the ramp and the step-ramp paradigms generated an increase in eye velocity starting ~100 ms after the onset of the target motion. In the ramp paradigm (left column) the gradual increase in eye velocity was interrupted by initial saccades (mean amplitudes: 2.01 deg for ME, 2.17 deg for MB) after which the eye velocity was close to the velocity of the stimulus. The mean gain during steady-state SPEM (i.e. late smooth pursuit that is stabilized by visual and motor feedback) was 1.02 for monkey MB and 0.94 for monkey ME during the time window of 300-500 ms after onset of stimulus motion. The latencies of initial saccades of the two monkeys were significantly different (t-test, t=93.51, df=7925, p<0.001); the average saccadic latency of monkey MB (Figure 4) was 217 ms (std = 41 ms), while for monkey ME (Figure 5) it was only 130 ms (std = 13 ms).

Compared to the saccadic latencies the average SPEM onset latency was very similar in both monkeys; 103 ms (std = 20 ms) for monkey MB and 107 ms (std = 21 ms) for monkey ME. This small difference between the monkeys, however, was highly significant (t-test, t=7.80, df=5921, p<0.001) due to the large number of trials. The SPEM onset is clearly visible for the horizontal eye velocity component in Figures 4 and 5 and also visible for the vertical component – in particular for large vertical angles of the target trajectory inducing larger vertical speed of the eyes (see magenta lines in Figures 4b and 5b). During the step-ramp paradigm (right column in Figures 4 and 5) the eye velocity increased until the stimulus velocity (10°/s) was reached approximately 200 ms after target motion onset. These general findings of the eye movements for the two paradigms are in good agreement with eye movements measured under similar conditions in humans (41, 46, 47).

**Figure 6:**
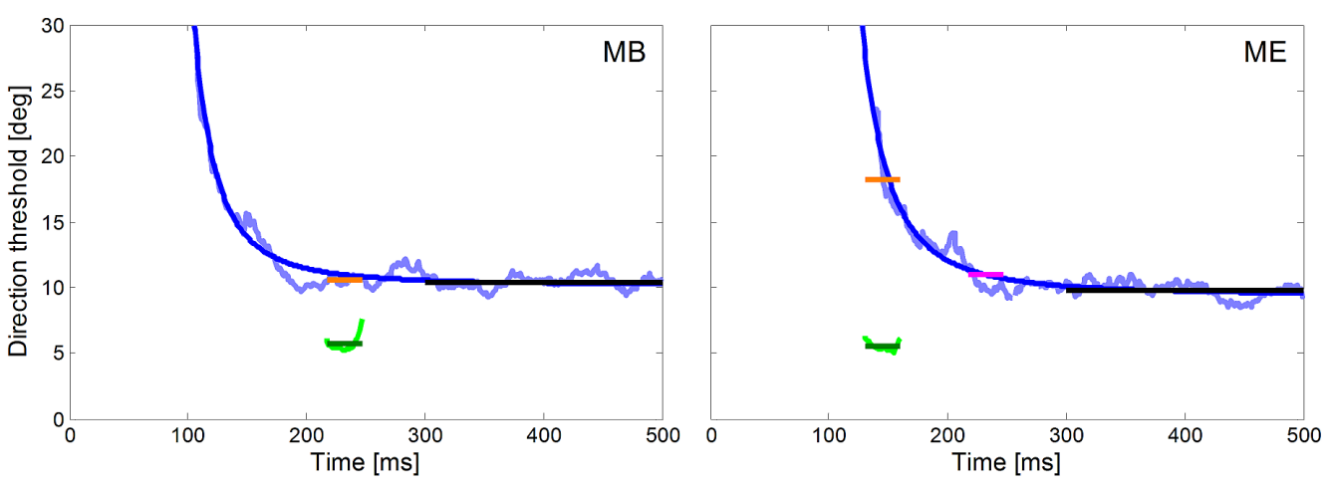
Time courses of direction thresholds of smooth pursuit and initial saccades for monkey MB (left) and ME (right) with respect to target motion onset. The direction thresholds for pure pursuit measured with the step-ramp paradigm are plotted in light blue and the fitted decay functions (Equation 1) in dark blue. Direction thresholds for initial saccades measured with the ramp paradigm are plotted in green for a 30 ms time window starting at saccade onset. This plot allows the comparison of the directional thresholds of saccades (green) and pursuit (STEP, orange) during the same time-window relative to the onset of target motion. For monkey ME, we marked in magenta the directional pursuit thresholds after additional 80 ms which corresponds to the saccadic reaction time of monkey MB. The average directional thresholds during steady-state SPEM (black lines) were calculated from 300 ms to 500 ms after the onset of stimulus motion.

For both monkeys, we also compared the directional precision of their initial saccades measured with the ramp paradigm with the average direction thresholds of pursuit during the step-ramp paradigm. The peri-saccadic direction thresholds were averaged in a 30 ms time window beginning with the saccade onset (green lines in Figure 6). We used the same time window during pure SPEM in the step-ramp paradigm. In the following we label the direction thresholds reached during pure SPEM measured at the time equivalent to the time of saccades the ‘Saccade Time Equivalent Pursuit thresholds’ (STEP). The averaged values of STEP are shown as orange lines in the fitted decay functions in Figure 6. Average direction thresholds during steady-state SPEM were calculated in a time window between 300 ms and 500 ms after the target motion onset (black lines in Figure 6). The numeric values of the direction thresholds in the different time windows are also shown in Table 1. It is obvious that the peri-saccadic thresholds of about 5 deg in both monkeys are lower by 5 deg for monkey MB and more than 10 deg for monkey ME than the corresponding STEP. The difference was larger in monkey ME because his saccadic latencies were more than 80 ms shorter than in monkey MB. Since the time course of precision follows a decay function, short latencies result in higher directional thresholds for SPEM. For monkey ME, the differences in STEP and peri-saccadic precision were similar to MB when the same time window (starting at 217 ms) was used for calculation of STEP in both monkeys (magenta line in Figure 6).

**Table 1:** Means and standard deviations of direction thresholds (in degrees) for monkeys MB and ME during saccades (Sacc), the same time window during SPEM initiation (STEP), and steady state pursuit.

Monkey	Sacc	STEP	Steady state
MB	5.7°/0.6°	10.6°/0.2°	10.4°/0.5°
ME	5.5°/0.3°	18.2°/2.6°	9.8°/0.6°

The temporal profile of the directional precision of pursuit during the step-ramp paradigm in man and monkey can be described by an exponential decay function (see Equation 1). One aim of our study was to compare the precision of the eye movements of the head unrestrained NHPs with previously published results obtained from human subjects (29, 30). In Figure 6 we show the time course of the directional thresholds in the step-ramp paradigm for the two monkeys (light blue lines) and the corresponding fits (dark blue lines). For both monkeys, we found a good approximation of the data by the decay function (time constant x3 = 587 ms, time to reach double asymptote = 121 ms, MSE = 0.52 for monkey MB, time constant x3 = 707 ms, time to reach double asymptote = 150 ms, MSE = 0.48 for monkey ME.

We found that although the general shape of the temporal profile of directional precision in our two NHPs was very similar to the results found in human subjects (e.g. 30), however the thresholds were overall higher in the NHPs than in human subjects. The possible reasons for this difference will be discussed later. In order to better compare the performance from humans and NHPs beyond this general difference we normalized the individual precision data by dividing it by the thresholds obtained during steady-state SPEM.

**Figure 7:**
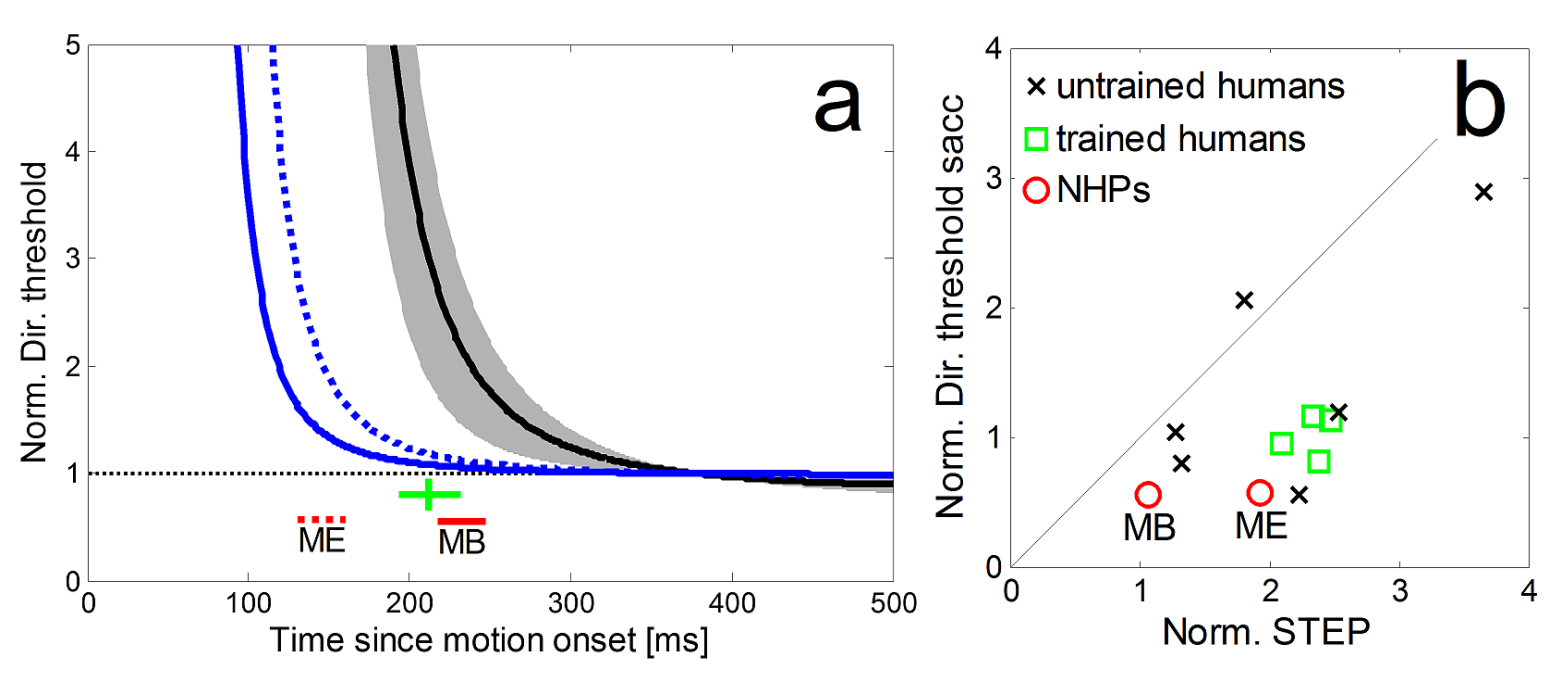
a: Comparison of the normalized time courses of directional precision in the step-ramp paradigm for two monkeys (blue lines) and the average of four trained human subjects (black line, grey area shows std) from Braun & Gegenfurtner (30). The corresponding peri-saccadic precisions and saccadic latencies are shown as red lines for the NHPs (dashed: monkey ME; solid: monkey MB) and a green cross (showing the average latency of the saccades as well as the mean and std of peri-saccadic direction thresholds) for the human subjects. b: Direction thresholds during the initial saccade in the ramp-paradigm and the corresponding times during SPEM-onset in the step-ramp paradigm (STEP) for monkey MB and ME (red circles). Thresholds were normalized by the asymptotic pursuit threshold. For comparison, data from four trained human subjects (green squares) and six untrained human subjects (black crosses) from Braun & Gegenfurtner (30).

The normalized time courses of directional precision in the step-ramp paradigm and the corresponding peri-saccadic precisions are shown in Figure 7a for our two monkeys (blue lines) in comparison to the average of four trained human subjects (black line) from Braun & Gegenfurtner (30). It shows a substantial difference in latency of the eye movements between NHPs and humans which is consistent with earlier reports (46). In agreement with the human data we also observed in NHPs that their peri-saccadic direction thresholds (Figure 7a red lines for NHPs, green lines for human subjects) were lower than the direction thresholds for pure SPEM during the same time relative to the onset of the stimulus motion. For both, humans and monkeys, the peri-saccadic thresholds were also lower than those during steady-state SPEM. In Figure 7b we compare the normalized peri-saccadic thresholds and STEP from our NHPs to data obtained from four trained and six naïve subjects (from 30). For both subject groups, STEPs were higher than peri-saccadic precision thresholds. The normalized peri-saccadic thresholds of our two NHPs appear slightly lower than those of human subjects but the sample-size is too small for a meaningful statistic.

## Discussion

We compared the oculomotor precision of initial saccades and SPEM of two head unrestrained macaque monkeys. While there were differences with respect to absolute precision values, we found good agreement in the temporal evolution of directional precision as well as in the relative differences in precision during initial saccades and SPEM initiation compared to humans (30). Both of our monkey subjects showed a lower precision compared to humans in the same experimental paradigms, especially during pursuit. This indicates that while we can reproduce the patterns of oculomotor behavior in humans and head-unrestrained NHPs, we also have to take the possibility of differences in absolute values into account. At first glance, this would limit the feasibility to directly combine data from humans and NHPs. As shown above, however, such combination is possible with normalized results from humans and NHPs. This is an important finding since the head-unrestrained approach as employed in our current study on monkeys is more similar to typical approaches in human oculomotor studies than with head-fixed monkeys.

### Comparison of direction precision during SPEM and saccades

Our results largely confirm previous reports on human oculomotor behavior showing that during saccades directional precision is higher than during SPEM (30). This is the case in particular when the peri-saccadic directional precision is compared to the Saccade Time Equivalent Pursuit thresholds (STEP) – these are the SPEM thresholds that were measured at the same time relative to the onset of stimulus motion. The degree to which saccadic direction thresholds were lower than STEP was dependent on the latency of the saccades. In our current study, the saccade latency of monkey ME was particularly short (130 ms, Figure 5) which resulted in particularly high STEP since the time-course of the precision follows a decay function. Since the peri-saccadic thresholds did not seem to be dependent on the saccadic latency (in (30), the correlation between saccade latency and peri-saccadic direction threshold was not significant) the difference between STEP and saccadic thresholds was much higher in ME than MB (for illustration see Figure 6). The conclusion for the human subjects as well as for head unrestrained monkeys is that the saccadic system receives quite accurate directional input very early so even saccades with very short latencies show low directional thresholds. In contrast, the SPEM-system either accumulates directional information over longer periods of time or is slower in translating the accurate sensory information in an equally accurate motor representation.

### Comparison to earlier studies of directional precision in NHPs

Our results largely confirm data of Osborne et al. (7) on the dynamics of directional precision during pursuit initiation in head-fixed monkeys. We found a similar overall time course of decreasing directional thresholds after pursuit initiation (compare our data shown in Figure 6 with Figure 10 A of (7). However, the absolute values of directional precision were different as the directional SPEM thresholds of the six monkeys in Osborne’s study were considerably lower. This difference in results probably originates from several sources. Firstly, and most importantly, Osborne and colleagues employed a head-fixed preparation, while we used a head-unrestrained approach. This was done on purpose since we aimed to employ an approach as similar as possible with typical human oculomotor studies. Since we measured eye-in-head-position rather than gaze direction, a part of the increased thresholds observed in our study might be caused by eye movements intended to compensate for (unregistered) head-movements.

Secondly, during our measurements we observed some noise in the eye position signal that was most likely a property of the experimental apparatus than of the monkey’s oculomotor behavior. It may have been caused by a relatively large distance between camera and eye as necessitated by our experimental setup. This noise obviously induced a lower absolute precision in our study compared to Osborne et al. (7).

A third difference between our and Osborne’s study was the apparatus used for the measurement of eye position. Osborne and colleagues (7) employed an invasive approach, i.e. they used implanted scleral search coils that are considered to be the gold standard regarding the precision of eye movement measurements. In our study, we employed a non-invasive approach, i.e. we used an infrared, video based eye-tracker (EyeLink 1000 Plus), as in Braun and Gegenfurtner (30). The accuracy and precision of these video-based eye trackers are potentially also very high (average accuracy ~0.5° as described in the manual) but also more dependent on the specifics of the experimental setup. Kimmel et al. (48) monitored simultaneously in two macaque monkeys the eye position with a sclera-embedded search coil and an optical tracker (Eyelink 1000) while they performed simple eye movement tasks, i.e. saccades and fixation but not SPEM. Their comparison of the two eye tracking techniques revealed a broad agreement and correlation in eye position, but also differences such as higher peak velocities for saccades and stronger post-saccadic oscillations for the optical eye-tracker.

### Performance differences between humans and NHPs

The neural substrate for the generation of eye movements involves largely the same processing stages in humans and macaque monkeys (e.g. 49). However, the exact properties like the latency, selectivity and sensitivity of the neurons at each of these stages may be different between the species reflecting anatomical constraints and ecological demands.

Studies that have compared oculomotor behavior of monkeys and humans (e.g. 46, 47, 50, 51, 52) often concluded that there is a ‘qualitative similarity’ between the results from humans and monkeys. This term generally means that the overall structure of oculomotor behavior and types of eye movements (e.g. catch-up saccades, express-saccades, pursuit initiation, steady-state SPEM) can be found in both species, although their specific parameters and absolute values (like latencies, peak velocities and precision) often differ. These discrepancies can be partly attributed to different neuronal substrates that allow monkeys to make eye movements with shorter latencies (47) and higher peak velocities (46) than humans. While we believe that this interpretation is reasonable in cases where monkeys outperform even well trained and motivated human subjects, we also think that the situation is not as clear cut in situations where monkeys show a degraded performance compared to humans as it is the case with our monkeys when compared to the results from Braun & Gegenfurtner (30). Human and monkey subjects may differ, e.g. in the degree of training in oculomotor tasks in preference between speed and accuracy or in motivation. While both of our monkey subjects were involved in similar tasks for a longer period of time, they were never forced to perform as precisely as possible. Hence, their preference may have been rather on speed than on accuracy. This, however, can’t fully explain the different performance before they reached steady-state pursuit since speed likely doesn’t play a major role in this late pursuit component. Bourrelly et al. (53) showed in NHPs how by training the quality of pursuit (eye velocity, gain) evolved and improved while the accuracy of interceptive saccades showed no difference.

NHPs in the experimental setting often show large inter-individual differences in performance as well as performances that are vastly inferior to performance of human subject under similar conditions. As an example, Liu & Newsome (54) found that the speed discrimination thresholds of two trained monkeys differed by a factor of 2 and did not change throughout the training period.

### Consequences for combining human and NHP data

Our results support the observation made in a number of studies, i.e. that oculomotor behavior from humans and NHPs shows the same general patterns but not always the same absolute values of the investigated parameters (46, 47, 50, 51, 52, 53). To make matters more complicated the oculomotor behavior of NHPs may show a superior performance in some aspects, e.g. saccadic latency, but inferior performance in others, e.g. precision. Accordingly, it is not always easily possible to predict the behavior of NHPs based on human data. Thus, caution should be applied when behavioral data from humans and electrophysiological recordings from NHPs are directly combined. The human data can be used as a source of information about what kinds of phenomena should be expected in NHPs, however, to link behavior directly to measurements of the underlying neurophysiological substrate in NHPs it still is required to investigate the behavior of NHPs directly.

In summary, while our findings in general support the feasibility of the monkey model for SPEM studies there are restrictions that can arise from subtle differences in the neural substrate as well as in differences in the experimental conditions as reported here. This should be kept in mind while modelling predictions of human behavior based on neuronal activities recorded in NHPs.

## Ethics and Conflict of Interest

The authors declare that the contents of the article are in agreement with the ethics described in http://biblio.unibe.ch/portale/elibrary/BOP/jemr/ethics.html and that there is no conflict of interest regarding the publication of this paper.

## Acknowledgements

This research was supported by SFB/TRR 135/ A1 and FOR 1847/A2.

We thank Andre Kaminiarz, Alexander Platzner and Katharina Martin for help with the hardware of the experimental apparatus (AP), animal care (KM), supervision of animal care and management of the NHP-facility (AK).
